# Supplementary material for: Charge carrier-selective contacts for nanowire solar cells
Source: Nat Commun. 2018 Aug 14;9:3248. doi: 10.1038/s41467-018-05453-5 (PMC6092389; doi:10.1038/s41467-018-05453-5)
Supplement: Supplementary file 1 — Supplementary Information [file 41467_2018_5453_MOESM1_ESM.pdf]

Supplementary Information for

Charge Carrier-Selective Contacts for Nanowire Solar Cells

Sebastian Z. Oener[1,3†], Alessandro Cavalli[2], Hongyu Sun[3], Jos E.M. Haverkort[2], Erik P.A.M. Bakkers[2,4], Erik C. Garnett[3†]

[1]Department of Chemistry and Biochemistry, University of Oregon, Eugene, Oregon 97403, USA.

[2]Applied Physics, Eindhoven University of Technology, PO Box 513, 5600 MB Eindhoven, The Netherlands.

[3]Center for Nanophotonics, AMOLF, Science Park 104, 1098 XG Amsterdam, The Netherlands

[4]Kavli Institute of Nanoscience, Delft University of Technology, Delft, The Netherlands

[†]To whom correspondence should be addressed. E-mail: garnett@amolf.nl, szo@uoregon.edu

Supplementary Figures

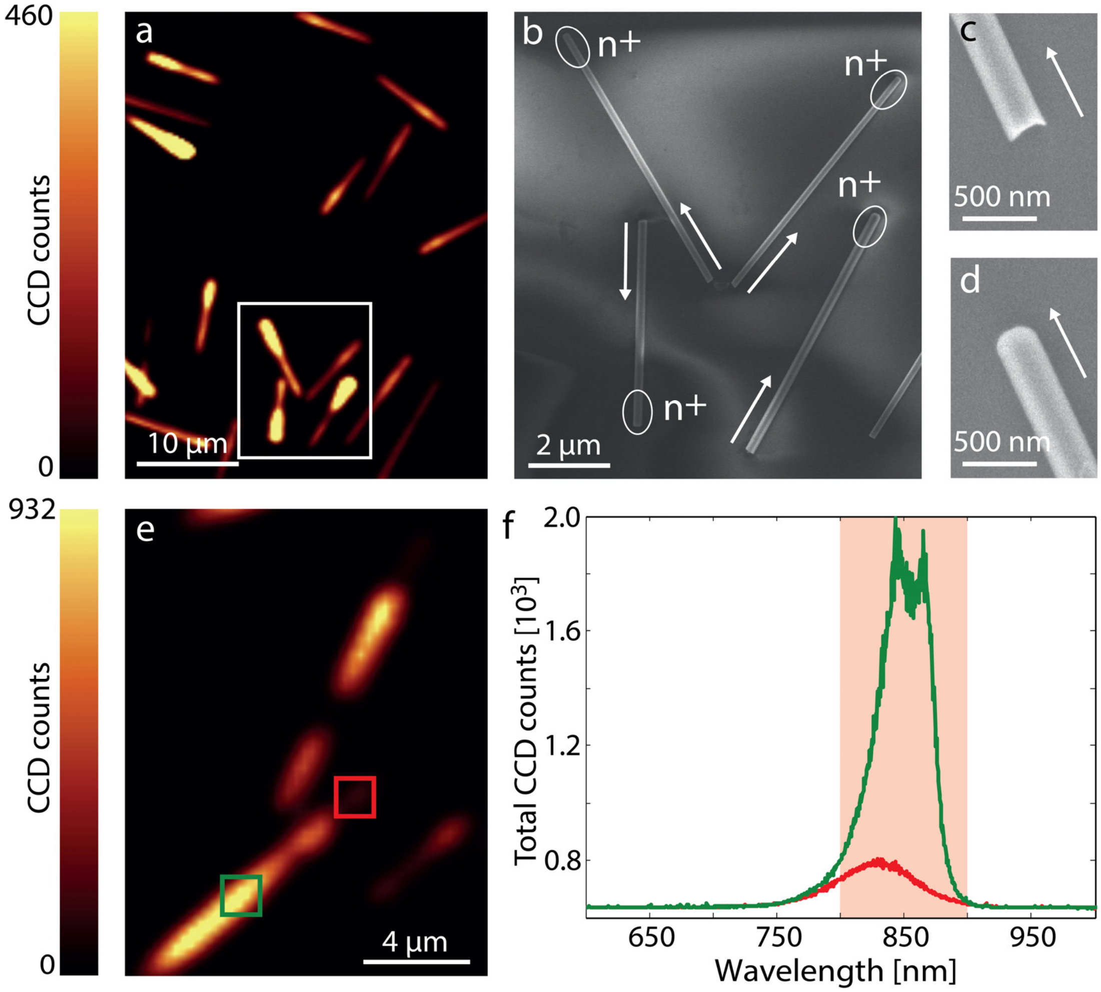

Supplementary Figure 1 | Variation in photoluminescence for randomly dropcast nanowires. (a) A map of PL intensity variations between different wires, all grown under the nominal same conditions. (b) SEM image of the marked region of (a). The SEM allows for localization of the broken end part and hence the orientation of the wires (white arrows in (c) and (d)). Together with the knowledge of the growth recipe (*p*-*i*-*n*, bottom-center-top), the bright end parts of the wires in (a) can be linked to the highly n^--doped region. Notably, for wires with similar diameters ($\Delta d \pm 10$ nm), the end parts as the rest of the wires do not show homogeneous brightness. (e) Strong PL variations along the length and between two different wires. (f) PL spectra of the two marked regions in (e). The shift in wavelengths and intensity due to the different doping concentration is clearly visible (explanation see text). The spectra have been averaged over the marked area from 800 nm to 900 nm to display the maps in (a) and (e).

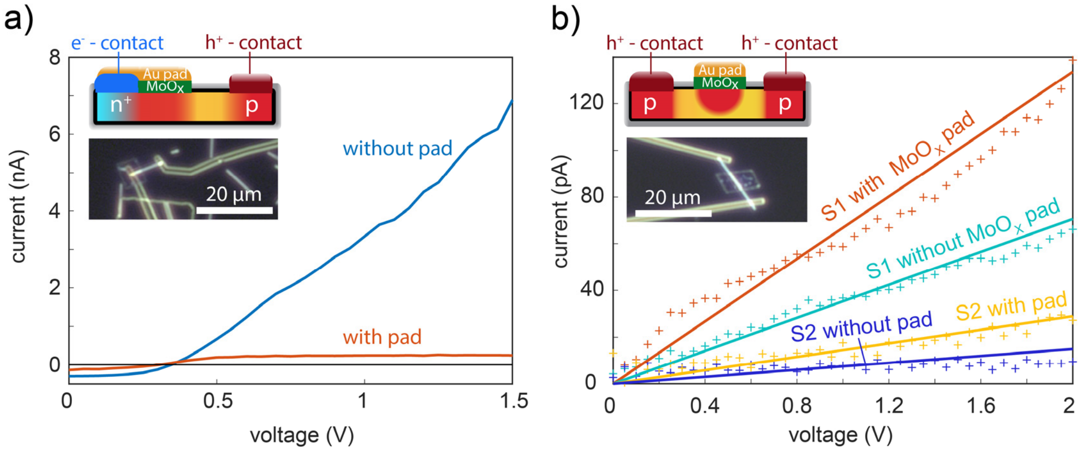

Supplementary Figure 2 | *I-V* curves for MoO_X pads located on differently doped segments. (a) For the rectifying doping geometry, *n-i-p*, a decrease in performance occurs when the MoO_X is placed on the *n*-doped segment of the wire, due to a decrease in electron conductivity. This behavior is in stark contrast to the case when MoO_X is placed on the *p*-type part, which increases the hole selectivity, as shown in the main text in Figure 2. (b) For a MoO_X pad that is centered on a wire with the symmetric doping profile *p-i-p*, the MoO_X increases the conductivity of the central nanowire part below it due to accumulation of holes. While the conductivity increase with MoO_X surface modification is clearly visible, the overall magnitude is limited. We ascribe this effect to the overall resistive behavior of the nanowires in the dark (see also Supplementary Figure 4). The result of two samples (S1 and S2) are shown. The lines are guides for the eye. The insets in (a) and (b) show a schematic of the geometry and an optical dark field image of a representative wire.

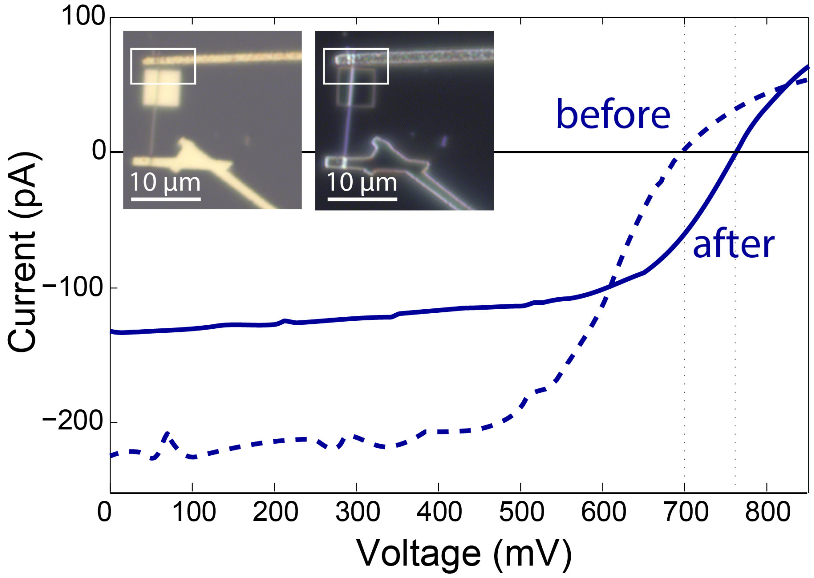

Supplementary Figure 3 | *I*-*V* curves of a nanowire with visibly disconnected MoO_X-Au pad. The *I*-*V* curves show a clear increase in V_{OC} with the application of the MoO_X pad (capped with Au) even though the pad is clearly disconnected from the ohmic metal contacts at the wire ends (bright- and dark-field optical microscopy images in the inset). Those results are in line with the observation of strongly resistive *I*-*V* curves for wires that were attempted to be connected only with MoO_X pads, i.e. without the ohmic contacts at the wire ends as shown for the wire here. Therefore, we conclude that the MoO_X surface layer increases the hole accumulation and hence selectivity inside the wire but does not facilitate charge carrier extraction, in contrast to traditional heterojunction interfaces.

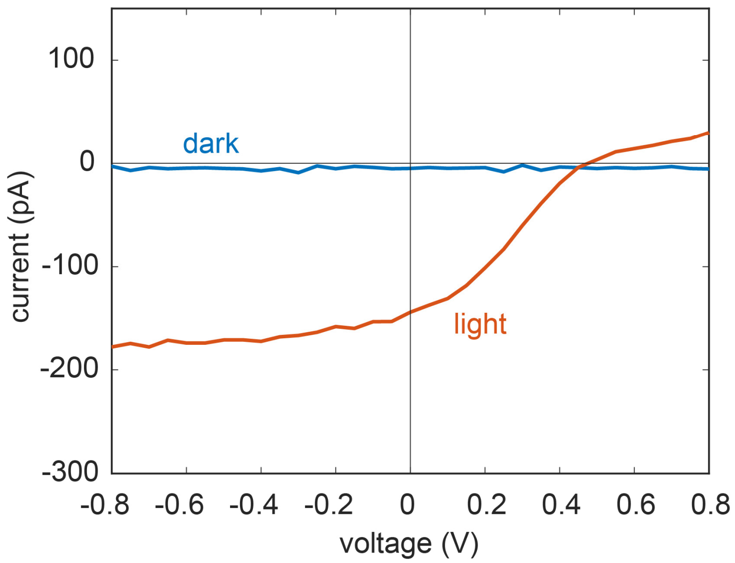

Supplementary Figure 4 | Representative *I-V* curves in the dark and in the light. We ascribe the resistive behavior of the nanowires in the dark (blue) to unintentionally low p-type doping (due to the mention complications of the *p*-type dopant Zn), the intrinsic segment in the center and the overall lengths of the nanowires (10 to 15 µm) required to study the influence of surface modifications. Under illumination (orange) the wires show the depicted behavior in the main text.

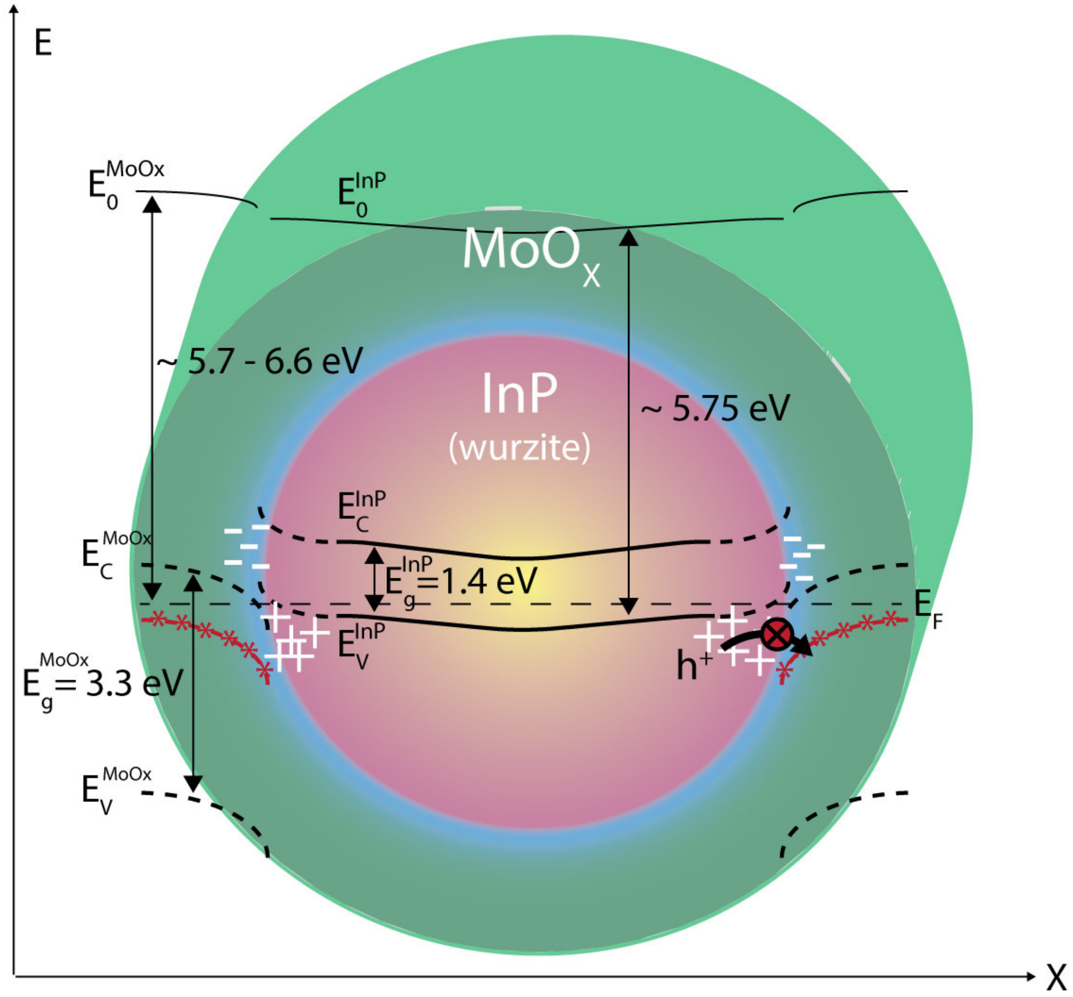

Supplementary Figure 5 | Schematic band alignment between InP and MoO_X in radial direction. For simplification the MoO_X (green) is shown in a wrapped-around gate geometry. While the Fermi level and band positions with respect to vacuum are known for the two materials in isolation, the exact band positions at the InP| MoO_X interface are unknown. From our experimental observations we must conclude that charge carrier extraction directly *via* the MoO_X is inhibited. The strong hole accumulation inside the InP and electron accumulation inside the MoO_X lead to strong band bending at the interface. As a result, holes have to move upwards in free energy (downwards in the energy diagram) to pass the extraction barrier.

Supplementary Note 1: Inhomogeneous Photoluminescence Maps

One of the main challenges of InP nanowires, in fact III-V semiconductor materials in general, is the formation of the hole-selective contact, that is the highly *p*-type doped region. The most widely used and also here employed *p*-dopant Zn shows strongly limited incorporation dynamics during growth, strongly increasing diffusion constants with increasing concentration[1–3] and is known to even cause increased non-radiative recombination.[4] Therefore, the formation of short (smaller than 500 nm) highly doped *p*-type nanowire segments with an abrupt doping profile is very challenging.[5,6] In view of those challenges it is understandable that the highest open-circuit voltages for InP nanowires so far (about 900 mV) have been obtained for geometries that utilize a highly Zn-doped substrate in direct contact to the highly p-type doped segment of the wire during growth.[7–9]

For nanowires, doping inhomogeneities can be visualized directly e.g. *via* atom probe tomography[10] or via the collection of luminescence after initial photon absorption, *i.e.* photoluminescence.[11,12] The absorption depends on the material geometry and the absorption coefficient and leads to the excitation of charge carriers. Subsequently, those carriers decay either radiatively or non-radiatively. Therefore, the photo-excited charge carrier density (determined by the absorption), the doping concentration and non-radiative recombination are essential. If structures are investigated that have the same geometric dimensions and are of the same material quality, photoluminescence can give detailed information about the doping density.[11,12]

Supplementary Figure 1a shows variations in photoluminescence (PL) for randomly dropcast nanowires that are indicative of variations in the doping profile. Supplementary Figure 1b shows an SEM image of the marked region of Supplementary Figure 1a. The SEM allows for localization of the broken bottom part and hence the orientation of the wires (white arrows in Supplementary Figure 1c and d). Together with the knowledge of the doping profile (*p*-*i*-*n*, bottom-center-top), the bright end parts of the wires in Supplementary Figure 1(a) can be linked to the highly n^--doped region. Furthermore, we conducted PL measurements before and after the contacting procedure for single nanowire devices. We employed specific contacts for the *n*-type (Ti/Au) and for the *p*-type part of the wire (Cr/Zn/Au) which allowed us to confirm our assumption about the orientation of the wire. For nanowires that have been oriented with the *p*-type part towards the electron contact (connected backwards), high extraction barriers have been observed. As can be clearly seen in Supplementary Figure 1a, the end parts, as the rest of the wires, do not show homogeneous PL brightness. Additionally, Supplementary Figure 1eshows strong PL variations not only between but also along the length of two different wires. The PL variations in Supplementary Figure 1a and Supplementary Figure 1eare noteworthy. The wires have very similar geometric dimensions ($\Delta d \pm 10$ nm) and hence a very similar absorption. Furthermore, detailed TEM analysis, as shown previously (Supplementary Information of Ref 3),[13] shows a high crystal quality, except for a 500 nm short section of the highly doped end part, which exhibits pronounced stacking faults.

Therefore, the absorption and non-radiative recombination due to defects can be assumed to be very similar across individual nanowires. Consequently, the PL variations that we observed can be related to a largely varying doping density, even though all nanowires have been grown under the same conditions. Supplementary Figure 1f shows the PL spectra of two marked regions in Supplementary Figure 1e. It is worth noting, that not only the intensity changes, as described above, but also a shift and a change of the PL spectra occurs. The spectrum of the middle part of the wire (green) shows two distinct peaks at around 840 nm (1.48 eV) and 860 nm (1.44 eV) which is related to a splitting of the valence band for wurtzite InP nanowires by 40 meV.[14,15] The spectrum on the end part of the wire (red) shows only one peak at 840 nm. Considering, that the end parts of the wires are supposedly

highly doped, we assume that a blue shift of the spectrum occurs because of band filling of the lower band. However, given that the intensity of the end part is strongly reduced compared to the middle part, the high doping must be accompanied by a strong non-radiative decay, such as Auger recombination. It is also important to note, that compared to the rest of the wire, the highly doped n^{++} end part shows a mixed crystal phase (zincblende and wurtzite) as observed by us before.[13] This fact has to be considered when discussing the red shift in detail, which is however beyond the scope of this publication. The spectra have been averaged over the marked area from 800 nm to 900 nm to display the maps in Supplementary Figure 1a and e.

In summary, the strongly varying doping profiles in Supplementary Figure 1 clearly show the difficulties in controlling the exact doping profile and homogeneity during the growth. Those results underline the importance of finding alternatives to impurity doping and the need for single nanowire studies. Instead of relying on ensemble averages of arrays, which consist of millions of different wires, isolated studies of single nanowires enable better understanding of the intricate mechanisms.

Supplementary Note 2: Charge carrier extraction barriers

In the literature, charge carrier extraction barriers have been observed for MoO_X heterojunction interfaces, i.e. for an interface where the MoO_X not only induces the carrier selectivity but also facilitates the carrier extraction (see also discussion regarding the band alignment between InP and MoO_X below).[16] In our case, we observe charge carrier extraction barriers not only after MoO_X surface modification (*e.g.* in Figure 2b) but also before (*e.g.* in Supplementary Figure 2). Therefore, even though we postulate a strong extraction barrier at the MoO_X|InP interface (see discussion below) we do not ascribe the observed extraction barrier to this interface. In fact, our additional experiments with MoO_X-only contacts and the devices with pads disconnected from the ohmic metal contact clearly show carrier extraction along the length of the nanowire, i.e. not through the MoO_X itself. Therefore, we ascribe the observed s-shaped *I-V* curves to non-ideal effective doping profiles along the longitudinal hole extraction path causing also the resistive behavior in the dark (see below).

For the surface modified nanowires, holes first encounter the high effective doping concentration under the MoO_X surface gate, i.e. the very region which is responsible for the studied increase in carrier selectivity of the contact by suppressing the electron current into the same direction. However, after traversing the surface gated region along the length of the nanowire, the holes encounter a region with the as-grown carrier concentration under the ohmic metal contact. This effective concentration profile, metal|n^+|n|i|p^+|p|metal is a non-ideal situation (the ideal case would be metal|n^+|n|i|p|p^+|metal) and can explain the occurrence of extraction barriers. This is further supported by the fact that devices with a gap between the MoO_X pad and the metal contact typically had larger extraction barriers.

We note, that all of those issues mentioned here are related to the specific horizontal single nanowire device geometry used to study the effect. We stress that with an ideal transparent wrap-around geometry and ohmic contact formation only on the very nanowire tip those issues would be prevented.

Supplementary Note 3: I-V curves in the dark and in the light

The resistive dark *I-V* curves (representative sample shown in Supplementary Figure 4) support the occurrence of charge carrier extraction barriers. The non-ideal doping profile (mainly due to the

mentioned issues regarding the *p*-type dopant Zn) includes resistive segments even limiting the current under illumination. We note that the observed increase in carrier selectivity of the *p*-type part in Figure 2 by a MoO_X pad covered by an opaque Au layer cannot be ascribed to an simple decrease in minority carrier concentration and hence conductivity due to the shadowing effect of the Au. The MoO_X + Au modifications on the *n*-type parts (Supplementary Figure 2) entailing a strong decrease in selectivity and conductivity (also in diode-forward direction!) clearly show the distinct effect of the high work function of MoO_X over a simple shadowing mechanism.

Supplementary Note 4: Band alignment between InP and MoO_X

Based on our experimental results we must conclude that hole extraction from the InP directly via the MoO_X surface layer is inhibited. Therefore, we speculate that a charge carrier extraction barrier must be present between the wurzite InP and MoO_X due to the specific band alignment between the two materials. Supplementary Figure 5 shows the proposed band diagram in the dark (and under illumination under the Au contact pad). The work function of MoO_X has been reported to lie between about 5.7 eV to 6.6 eV, depending on purity (e.g. decreasing with increasing carbon content).[17] Furthermore, MoO_X has been shown to essentially act as a high work function metal (5.75 to 6.70 eV) with a defect band right under the Fermi level (red lines in Supplementary Figure 5) which facilitates hole transport even though the valence band of MoO_X lies much more negative than the valence bands of many semiconductors.[17] For wurtzite InP the valence band lies around 5.75 eV while the band gap is 1.43 eV (300 K).[18] Therefore, we speculate that during equilibration in the dark a strong hole accumulation layer in the InP and an electron accumulation layer inside the MoO_X are formed. The bands inside the InP are bend upwards and inside the MoO_X downwards at the interface. As a result, the hole transport defect band inside the MoO_X is moved downward as well, increasing the energy difference to the InP valence band. Holes that have to pass the barrier from either side have to move downwards in the energy diagram (their free energy increases); an energy barrier is established. This is a very similar situation as the one observed for the MoO_X|silicon interface which can be explained by a similar band misalignment (which however is not as extreme as here and still allows for carrier extraction). [16]

We stress the schematic nature of Supplementary Figure 5. In particular, the degree of inversion inside the nanowire might be more pronounced, leading to a stronger effective external doping of the InP. The overall measured V_{OC} has to be considered as sort of average over the local V_{OC} with increasing contributions of regions with higher minority carrier conductivity (essentially loss channels of free energy). Therefore, to explain the observed increase in effective *p*-type doping the whole wire volume wire must see a substantial accumulation.

Supplementary Methods

Photoluminescence Maps

The photoluminescence spectra were obtained with a Witec confocal microscope (Alpha 300 SR). The samples were illuminated with a 532 nm laser, and the light was focused and collected via a 100X NA0.9 objective. A spectrometer with a 150 g mm^{-1} (BLZ= 500 nm) grating (center wavelength 760 nm) and a cooled CCD camera have been used. The integration time was 0.09 s and the scan speed 6.83 s per line.

Supplementary References

1. Logan, R. A., Chu, S. N. G., Geva, M., Ha, N. T. & Thurmond, C. D. Zinc incorporation into InP grown by atmospheric pressure metalorganic vapor phase epitaxy. *J. Appl. Phys.* **79,** 1371–1377 (1996).

2. Chu, S. N. G., Logan, R. A., Geva, M. & Ha, N. T. Concentration dependent ZN diffusion in InP during metalorganic vapor phase epitaxy. *J. Appl. Phys.* **78,** 3001–3007 (1995).

3. Chu, S. N. G., Logan, R. A., Geva, M., Ha, N. T. & Karlicek, R. F. Substitutional, interstitial, and neutral zinc incorporation into InP grown by atmospheric pressure metalorganic vapor phase epitaxy. *J. Appl. Phys.* **80,** 3221–3227 (1996).

4. Zhang, W. *et al.* Recombination dynamics in aerotaxy-grown Zn-doped GaAs nanowires. *Nanotechnology* **27,** 455704 (2016).

5. Wallentin, J. & Borgström, M. T. Doping of semiconductor nanowires. *J. Mater. Res.* **26,** 2142–2156 (2011).

6. Wallentin, J. *et al.* Degenerate p-doping of InP nanowires for large area tunnel diodes. *Appl. Phys. Lett.* **99,** 253105 (2011).

7. Wallentin, J. *et al.* InP Nanowire Array Solar Cells Achieving 13.8% Efficiency by Exceeding the Ray Optics Limit. *Science* **339,** 1057–1060 (2013).

8. Zhong, Z. *et al.* Efficiency enhancement of axial junction InP single nanowire solar cells by dielectric coating. *Nano Energy* **28,** 106–114 (2016).

9. van Dam, D. *et al.* High-Efficiency Nanowire Solar Cells with Omnidirectionally Enhanced Absorption Due to Self-Aligned Indium–Tin–Oxide Mie Scatterers. *ACS Nano* **10,** 11414–11419 (2016).

10. Perea, D. E. *et al.* Direct measurement of dopant distribution in an individual vapour-liquid-solid nanowire. *Nat. Nanotechnol.* **4,** 315–319 (2009).

11. van Weert, M. H. M. *et al.* Large redshift in photoluminescence of p-doped InP nanowires induced by Fermi-level pinning. *Appl. Phys. Lett.* **88,** 043109 (2006).

12. Wang, F. *et al.* Spatially Resolved Doping Concentration and Nonradiative Lifetime Profiles in Single Si-Doped InP Nanowires Using Photoluminescence Mapping. *Nano Lett.* **15,** 3017–3023 (2015).

13. Mann, S. A. *et al.* Quantifying losses and thermodynamic limits in nanophotonic solar cells. *Nat. Nanotechnol.* **11,** 1071–1075 (2016).

14. Tuin, G. L. *et al.* Valence band splitting in wurtzite InP nanowires observed by photoluminescence and photoluminescence excitation spectroscopy. *Nano Res.* **4,** 159–163 (2011).

15. De Luca, M. *et al.* Polarized Light Absorption in Wurtzite InP Nanowire Ensembles. *Nano Lett.* **15,** 998–1005 (2015).

16. Battaglia, C. *et al.* Silicon heterojunction solar cell with passivated hole selective MoO x contact. *Appl. Phys. Lett.* **104,** 113902 (2014).

17. Battaglia, C. *et al.* Hole Selective MoO x Contact for Silicon Solar Cells. *Nano Lett.* **14,** 967–971 (2014).

18. Jackson, H. E. *et al.* Probing the valence band structure of wurtzite InP nanowires by photoluminescence excitation spectroscopy. in *AIP Conf. Proc.* **1399,** 481–482 (2011).
